# Treating posttraumatic stress disorder in substance use disorder patients with co-occurring posttraumatic stress disorder: study protocol for a randomized controlled trial to compare the effectiveness of different types and timings of treatment

**DOI:** 10.1186/s12888-021-03366-0

**Published:** 2021-09-07

**Authors:** Sera A. Lortye, Joanne P. Will, Loes A. Marquenie, Anna E. Goudriaan, Arnoud Arntz, Marleen M. de Waal

**Affiliations:** 1 Arkin Mental Health Care, Jellinek, Amsterdam Institute for Addiction Research, Amsterdam, The Netherlands; 2 Amsterdam UMC, Department of Psychiatry, University of Amsterdam, Amsterdam, The Netherlands; 3 Amsterdam Public Health Research Institute, Amsterdam, The Netherlands; 4 Department of Clinical Psychology, University of Amsterdam, Amsterdam, The Netherlands

**Keywords:** Post-traumatic stress disorder, Substance use disorder, Prolonged exposure, Eye movement desensitization and reprocessing, Imagery rescripting, Treatment timing, Simultaneous, Sequential, Trauma-focused treatment

## Abstract

**Background:**

Posttraumatic stress disorder (PTSD) and substance use disorder (SUD) have high comorbidity. Although prior research indicated that PTSD can effectively be treated with Prolonged Exposure (PE) in these patients, reported effects are small and treatment dropout rates high. Eye Movement Desensitization and Reprocessing (EMDR) and Imagery Rescripting (ImRs) are other promising treatment options for PTSD, that have not yet been examined in this patient group. Furthermore, it is unclear whether PTSD treatment is most effective when offered simultaneous to or after SUD treatment.

**Methods:**

In this article, the Treatment Of PTSD and Addiction (TOPA) study is described: a Dutch randomized controlled trial (RCT) that studies the effectiveness of PTSD treatment as an add-on to regular SUD treatment in patients with SUD and co-occurring PTSD. Effects of PE, EMDR, ImRs, and a 3-month SUD treatment only condition will be compared, as well as simultaneous SUD/PTSD treatment to sequential SUD/PTSD treatment. The primary outcome measure is PTSD symptoms. Secondary outcomes are: treatment completion, psychological distress, substance use, interpersonal problems, emotion dysregulation, and trauma-related emotions guilt, shame, and anger.

**Discussion:**

This study is the first to compare effects of PE, EMDR, and ImRs in one study and to compare simultaneous SUD/PTSD treatment to sequential SUD/PTSD treatment as well. This RCT will provide more knowledge about the effectiveness of different treatment strategies for PTSD in patients with co-occurring SUD and will ultimately improve treatment outcomes for patients with this common co-morbidity worldwide.

**Trial registration:**

Netherlands Trial Register (NTR), Identifier: NL7885. Registered 22 July 2019.

## Background

### PTSD and SUD comorbidity

Posttraumatic stress disorder (PTSD) and substance use disorder (SUD) often co-occur, with an estimated prevalence of SUD amongst individuals with PTSD of 46% in an epidemiologic study in the United States [1]. Studies in patients with SUD have reported rates of current PTSD amongst individuals with SUD of 25 to 34% [2, 3], with the highest rates reported in SUD patients with both alcohol and drug use disorders [3]. There are different causal pathways that may explain this high co-occurrence, that are not mutually exclusive. Firstly, SUD could lead to an increased risk of developing PTSD by leading a more risky lifestyle, which increases chances to experience traumatic events (e.g. being assaulted violently or sexually when being under influence of substances) [4]. Secondly, several studies indicated that PTSD can lead to the development of SUD as people attempt to self-medicate PTSD symptoms by using substances (e.g. [4, 5]. Thirdly, the onset or maintenance of both SUD and PTSD could be related to a shared underlying factor such as genetic vulnerability [6, 7].

### PTSD treatment

Prolonged Exposure (PE) and Eye Movement Desensitization and Reprocessing (EMDR) are both first line treatments for PTSD that have been studied extensively and shown to be effective treatments for reducing PTSD symptoms [8], also in patients with severe co-occurring disorders such as psychotic disorders [9]. Unfortunately, despite the high prevalence, patients in treatment for SUD are often excluded from randomized controlled trials (RCTs) evaluating PTSD treatments [10]. However, several studies have been conducted to examine the effectiveness of PE specifically in patients with co-occurring PTSD and SUD. A Cochrane review indicated that trauma-focused exposure-based interventions, such as PE, have consistently been found to be effective in reducing PTSD symptoms in patients with SUDs, when added to regular addiction treatment [11]. However, trauma-focused exposure-based interventions are found to be less effective in individuals with PTSD and SUD compared to individuals with PTSD alone. This may be related to the finding that, when trauma-focused interventions are added to regular addiction treatment, treatment drop-out rates are higher than in regular addiction treatment [12]. Furthermore, clinicians perceive individuals with both disorders as more difficult to treat than individuals with either PTSD or SUD alone [13].

Hitherto it is unknown how treatment completion can be increased in this difficult to treat group of patients. In a recent RCT a 90-min trauma-focused motivational enhancement session was added prior to PE therapy in order to increase treatment completion and effectiveness of PE in patients with PTSD/SUD. Unfortunately, adding this session did not lead to better PE retention than PE alone [14].

In patients with co-occurring PTSD/SUD, the effectiveness of EMDR has been studied in only one randomized pilot study [15]. Although this study had a sample size of only 12 patients, it indicated that adding EMDR to regular addiction treatment leads to a significant reduction of PTSD symptoms [15]. However, further research with a larger sample size is essential to draw further conclusions on the effectiveness of EMDR in this patient group. Possibly, treatment drop-out rates may be lower in EMDR compared to PE, since EMDR requires only brief activation of exposure to the traumatic experiences instead of prolonged reliving of traumatic experiences. The same accounts for yet another promising treatment option, namely Imagery Rescripting (ImRs), although this treatment has never been studied in patients with both PTSD and SUD.

ImRs is a therapy that is becoming increasingly popular for treating PTSD and other disorders. A meta-analysis of 19 studies showed that ImRs is effective in reducing aversive imagery and related psychological complaints, with large effects obtained in a small number of sessions [16]. In chronic PTSD patients, the addition of ImRs to PE led to a significant reduction of treatment drop-outs and better effects on anger control, externalization of anger, hostility and guilt compared to PE alone [17]. Furthermore, ImRs was found to be effective in patients who did not respond well to PE and predominantly experienced non-fear emotions like shame, guilt and anger [18]. Two subsequent studies found that ImRs is effective as a stand-alone treatment for PTSD after childhood abuse [19, 20]. Furthermore, two meta analyses both indicated that ImRs has positive effects in the treatment of nightmare frequency, sleep quality and PTSD symptoms [21, 22]. A recent RCT directly compared ImRs to EMDR in patients with PTSD from childhood experiences and found that both treatments are equally effective [23].The release of the *Diagnostic and Statistical Manual of Mental Disorders 5* (DSM-5) [24] has expanded the scope of criteria of PTSD beyond a fear-based concept, considering that a traumatic event can also be followed by shame, guilt and/or anger. Trauma-related shame and guilt seem to play a central role in the maintenance of PTSD symptoms, by contributing to emotional aversiveness of the trauma memory. Higher degrees of shame and guilt in PTSD are associated with depression and anxiety symptoms [25], and with higher levels of PTSD symptoms through treatment [26]. A review illustrated that especially shame is a notable component of PTSD and that reducing shame may increase the effectiveness of the treatment of PTSD symptoms [27]. Individuals with a history of childhood trauma and neglect experience more intense feelings of shame and sadness than healthy controls without childhood abuse and neglect, whereas higher levels of shame and sadness are related to substance use [28]. This substance use is generally hypothesized to be an emotion regulation strategy, therefore childhood trauma may increase the risk for developing SUD at least partially through increased levels of shame and sadness [28]. Given the above described effectiveness in reducing drop-out and feelings of anger, shame and guilt in patients with PTSD, ImRs is a promising approach for treating PTSD in patients with SUDs.

In summary, although prior studies indicated PE is an effective PTSD treatment for patients with co-occurring PTSD and SUD, effects were small and treatment drop-out rates were high. EMDR and ImRs are other promising treatment options for PTSD, that have not yet been examined in this difficult to treat patient group. Furthermore, head-to-head comparisons of these active PTSD treatments are scarce, and completely lacking in SUD/PTSD patients.

### Treatment order

In most published studies in which a trauma-focused treatment was added to SUD treatment, it is unclear whether treatments for PTSD and SUD were delivered simultaneously (starting at the same time) or sequentially (starting PTSD treatment after finishing SUD treatment) [7, 29] A Dutch study showed that simultaneous PTSD/SUD treatment can be safely implemented in inpatient as well as outpatient addiction care without negative effects on SUD treatment [30]. Yet another study showed that delivering PE and SUD simultaneously did not lead to deterioration of PTSD or SUD symptoms. When looking at individual changes during therapy with change analyses instead of reliance on means, patients who did experience an increase of PTSD or SUD symptoms somewhere during treatment, still improved on these symptoms at the end of treatment [31]. Dutch guidelines recommend simultaneous treatment of PTSD and SUD [29], whereas international guidelines (e.g. APA; ISTSS; NICE) do not address the issue of treatment order. However, treatment facilities often promote sequential treatment in which PTSD is treated after SUD treatment is finished. A previous study indicated that some clinicians working in addiction facilities strongly argue against simultaneous treatment [32]. These clinicians report to have too limited time and resources to adequately treat PTSD and report to believe simultaneous treatment to be counterproductive and harmful by eliciting craving and relapse. In contrast, in a more recent vignette study among clinicians, most clinicians preferred simultaneous SUD and PTSD treatment, because they believe that PTSD complaints maintain the SUD. However, most clinicians indicated that at their own workplace they found it difficult to implement simultaneous treatment due to a limited amount of inpatient facilities as well as lack of expertise [33]. Recently, simultaneous treatment has been directly compared to phased treatment in an RCT [34]. In this study, the PTSD treatment consisted of PE whereas the SUD treatment consisted of Motivational Enhancement Therapy. In the phased treatment condition, the PTSD treatment started after 4 (out of 12) weekly sessions of SUD treatment. In contrast to the hypothesis, no differences in PTSD symptoms, SUD symptoms and treatment drop-out rates were found between the two groups [34].

Besides studies that have examined PTSD treatments that were added to SUD treatments, there are several studies that have examined the effectiveness of integrated treatments that integrate SUD and PTSD components within one treatment. Most consistent evidence is found for COPE (concurrent treatment of substance use disorders and PTSD using prolonged exposure), that includes motivational enhancement and CBT for SUD, psychoeducation relating to both disorders, and PE for PTSD [11, 35]. Another integrated treatment is Seeking Safety, a non-trauma-focused intervention that aims to reduce both PTSD and SUD by focusing on safe coping skills [36]. The previously mentioned Cochrane review found no improvement for PTSD severity when non-trauma-focused interventions were compared to usual care or to another active psychological therapy [11]. In summary, although guidelines recommend simultaneous treatment for PTSD and SUD, many therapists argue against this approach. Furthermore, studies directly comparing simultaneous with sequential treatment are lacking. More knowledge about this subject is necessary to improve treatment guidelines for co-occurring PTSD and SUD and enhance treatment outcomes of patients with this common comorbidity.

### Current study

This paper describes the study design of the Treatment Of PTSD and Addiction (TOPA) study, a Dutch RCT in patients with co-occurring PTSD and SUD who will receive PTSD treatment as an add-on to regular SUD treatment.

The primary objectives of this study are:

1. To compare effectiveness of PE, EMDR, and ImRs as add-on to regular SUD treatment with SUD treatment only in patients with co-occurring PTSD/SUD.
2. To compare effectiveness of simultaneous SUD/PTSD treatment with sequential SUD/PTSD treatment in patients with co-occurring PTSD/SUD.
3. To explore differential effectiveness between active PTSD treatments (PE vs. EMDR; PE vs. ImRs, EMDR vs. ImRs) in patients with co-occurring PTSD/SUD.

We expect that at 3-month follow-up, all trauma focused therapies will have led to a stronger reduction of the primary outcome PTSD symptoms than the SUD treatment only condition (objective 1). In addition, we expect that, compared to the SUD treatment only condition, all trauma focused therapies will have led to a stronger reduction of the following secondary outcomes: psychological distress, substance use, interpersonal problems, emotion dysregulation, guilt, shame and anger. We expect a greater reduction of PTSD symptoms in the simultaneous treatment condition compared to the sequential treatment condition at 6 and 9-month follow-up (objective 2). We have no specific hypothesis about the direction of the differences in effectiveness between the three active PTSD treatments (objective 3).

Due to the COVID-19 outbreak in March 2020, temporary changes in both treatment as well as assessments had to be made, as the regular face-to-face contacts were temporarily not allowed at the treatment facility center where this study is conducted. In this article the original design of the study is described in the method section. At the end of the method section, all adjustments that were made due to COVID-19 outbreak are described.

## Methods

### Design

**Fig. 1:**
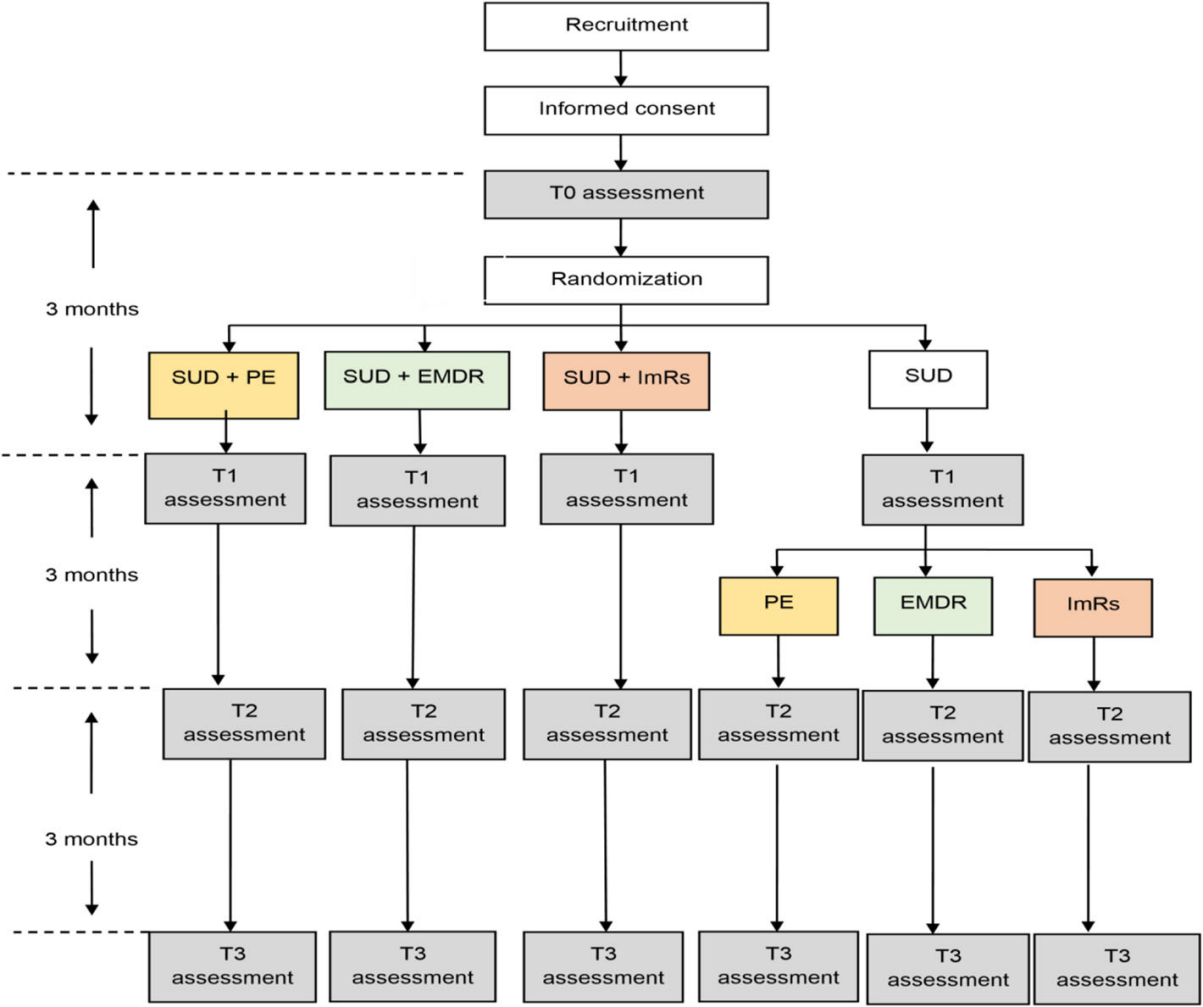
Flow chart of the study design. Abbreviations: SUD: Substance Use Disorder; PE: Prolonged Exposure; EMDR: Eye Movement Desensitization and Reprocessing; ImRs: Imagery Rescripting. Note T0–3 are subsequently the baseline assessment, and assessments at 3 month, 6 month and 9 month follow up

This study is a single blind 6-arm randomized controlled trial, consisting of 3 arms for simultaneous SUD/PTSD treatment with the PTSD treatments consisting of PE, EMDR, and ImRs, and 3 arms for sequential SUD/PTSD treatment with the PTSD treatments also consisting of PE, EMDR, and ImRs. Within the first 3 months after baseline, the three sequential treatment arms together form the SUD treatment only condition, as is depicted in the trial flow (Fig. 1). First, patients are randomly allocated to simultaneous PE, EMDR, or ImRs, or the SUD treatment only group. Subsequently, patients in this last group are randomly allocated to sequential PE, EMDR, or ImRs. The study is conducted at two departments of Jellinek. Jellinek is a Dutch addiction treatment facility center, offering both intramural and extramural addiction treatment. The duration of the study is planned to be 3.5 years, from inclusion of the first participant until the last follow-up measure of the final participant. The research protocol has been approved by the Medical Ethical Committee of the Amsterdam Academic Medical Centre (AMC) and the trial has been pre-registered at the Netherlands Trial Register (NTR L7885). The study is currently running.

### Participants

Participants are recruited at the Jellinek Center in Amsterdam and Utrecht. Study participants are patients who apply for SUD treatment at the addiction treatment center, for whom treatment of co-occurring PTSD is also indicated. All patients treated in the two participating locations are adults (18 years of age or older), and both males and females can participate in the study. The criteria for inclusion in the study are a) age 18 years or older; b) SUD(s) according to the DSM-5 (American Psychiatric Association, 2013), with a primary diagnosis involving one of the following substances: alcohol, cannabis, cocaine (snorting), amphetamine, benzodiazepine, opioid; c) PTSD according to the DSM-5 criteria; d) sufficient understanding of the Dutch language to be able to fill out Dutch questionnaires and follow therapy in Dutch. The exclusion criteria are: a) acute psychotic disorder; b) mental retardation or cognitive impairment (estimated IQ < 70); c) current physical or sexual abuse or death threats; d) current acute suicidal behavior (high suicide risk and suicide attempt in the last 3 months); e) life threatening self-injury; f) homelessness; g) involvement in a compensation case or legal procedures concerning admission or stay in the Netherlands, h) involvement in legal procedures regarding the index trauma, i) engagement in any other current PTSD treatment.

### Sample size

First, the required sample size was calculated to explore differential effectiveness between active PTSD treatments in reducing PTSD symptoms, regardless of timing of PTSD treatment. Three pairwise comparisons will be conducted between the active treatment arms (ImRs vs. PE, ImRs vs. EMDR; PE vs. EMDR). Due to the exploratory nature of this objective, an alpha of 0.05 and power of 0.80 are acceptable. Patients in the SUD treatment only condition will be randomly allocated to PE, EMDR or ImRs after SUD treatment and therefore are included in comparisons of the active treatment arms. To detect a medium effect size (d = 0.50) in a pairwise comparison between two active treatment arms, with alpha = 0.05, power = 0.80 and within-person correlation coefficient = 0.60, 52 participants are needed per arm. To make three pairwise comparisons 3 * 52 = 156 participants are needed for this comparison. Second, the required sample size was calculated to compare the timings of PTSD-treatment (simultaneous vs. sequential), regardless of type of PTSD-treatment. The allocation ratio between sequential and simultaneous PTSD treatment is 1:3. To be able to detect a medium effect size (d = 0.45) in a comparison between two arms, with an alpha = 0.05, power = 0.80 and within-person correlation coefficient = 0.60, 41 participants are needed in the sequential arm and 123 participants are needed in the simultaneous arm. In total, 41 + 123 = 164 participants are needed for this comparison. Finally, we expect 20% of the participants to drop out of the study, therefore in total 205 participants will be included in the study.

### Treatments

#### Treatment procedure

All participants receive regular SUD treatment plus either one of the following PTSD interventions: PE, EMDR or ImRs. SUD treatment starts directly after randomization for all participants. For patients allocated to the simultaneous treatment condition, PTSD treatment starts within 2 weeks after the start of the SUD treatment. For those in the sequential treatment condition, PTSD treatment starts 3 months after the start of the SUD treatment.

All PTSD treatments are provided by trained therapists and consist of twelve 90-min face-to-face sessions, conducted twice a week. Every treatment is executed by two different PTSD therapists, who alternately provide a session, inspired by the therapist rotation model. Among other things, this approach has shown to decrease avoidance behavior in sessions. Therefore, it is promising to improve trauma-focused treatment [37]. In all PTSD treatments, the focus of the first session is to present the treatment rationale and to list the traumatic experiences that will be addressed during the course of treatment. This overview is discussed and approved in peer-supervision after the first session. Henceforth the second session, each session contains 60 min of the specific intervention; EMDR, ImRs of PE. The rest of the session will be used to administer self-report questionnaires, to rehearse the rationale, to determine the specific trauma that will be addressed in the session and evaluation of the session. The self-report questionnaires are used to monitor symptom change during treatment. Although therapists encourage patients to be abstinent, this is not a requirement for the PTSD treatment. However, when a therapists notices a patients is intoxicated to such an extent that he/she has no capability to learn, the session is rescheduled.

#### PE

PE involves confrontation with distressing stimuli related to the trauma until anxiety is reduced. It is a manualized intervention including both imaginal and in vivo exposure components [38, 39]. Imaginal exposure uses confrontation to trauma memories through imagination, whereas in vivo exposure involves confronting real life situations that provoke anxiety and are avoided because of their association with the traumatic event. The aim of both is to extinguish the conditioned emotional response to stimuli associated with trauma [38]. Treatment sessions consist of 60 min of prolonged imaginal exposure (repeated recounting of the most anxiety provoking traumatic situations and related thoughts and feelings), and exposure in vivo (approaching trauma-related cues and situations). Between sessions, participants listen to audio recordings of the imaginal exposure on a daily basis, and complete in-vivo homework assignments. Within PE, only traumatic events that meet up the A-criterion of the DSM-5 can be included in the case conceptualization.

#### EMDR

EMDR is a manualized intervention, intended to reduce the liveliness of the traumatic memories and to change dysfunctional beliefs about self or others in terms of the traumatic event [40]. In the therapy the patient is asked to hold the distressing image in mind, along with the associated negative cognitions and bodily sensations, while engaging in saccadic eye movements. After approximately 20 s, the therapist asks the patient to note any changes occurring in the image, sensations, thoughts or emotions. This process is repeated until desensitization has occurred [40]. In this study the Dutch adaptation of Shapiro’s basic EMDR protocol is followed [41]. In this Dutch version, focus is more on reaching a high and optimal level of arousal instead of reassuring the patient, since high levels of emotional arousal have been found to enhance the memory-degrading effects of eye movements [42]. Therefore, the exercise of the safe place is absent in this version and rapidity of the procedures is higher. Furthermore, target selection is more extensively determined in the Dutch version [43]. Although traumatic events that meet the A-criterion of PTSD according DSM-5 will be addressed first, traumatic events that do not meet the A-criterion of the DSM-5 can also be addressed with EMDR, if there are still PTSD symptoms and sessions left.

#### ImRs

ImRs is a technique that changes the meaning of emotional memories and images (like intrusions and nightmares). With ImRs the individual is instructed to imagine the trauma memory as vividly as possible, as if it is happening in the here and now, and to imagine that the sequence of events is changed in a direction that the person desires [44]. The procedure aims to correct dysfunctional meanings attached to trauma memories and restore perceived control. The protocol is based on the ImRs protocol described by Arntz and Weertman [45]. In a minimum of two and a maximum of 5 sessions after the first session, the therapist steps into the image and rescripts, the patient staying in the perspective of their former self. After this, patients themselves rescript by stepping in the image from the perspective of their current self and intervening to protect and meet up with the needs of their former self. After completing this imagination of a new script, the patient experiences the new script from the perspective of their former self, during which they can ask for additional actions by their present self. The procedure of rescripting continues until the patient is satisfied. As with EMDR, traumatic events that do not meet the A-criterion of DSM-5 traumas can be addressed with ImRs, if there are still PTSD symptoms and sessions left.

#### Treatment completion and early responders

PTSD treatment completion is defined as a participant completing all 12 sessions of PTSD treatment within the allocated timeframe or being an early responder. For the simultaneous treatment condition, the timeframe for PTSD treatment is between baseline and 3-month follow-up. For the sequential treatment condition, the timeframe for the PTSD treatment is between 3-month and 6-month follow-up. Non-completers or drop-outs are participants who do not complete their PTSD treatment within the allocated timeframe. Participants who do not show for one of more treatment session are contacted by their therapists and motivated to commit to the treatment. Treatment is halted when a participant decides to stop or when the end of the timeframe is reached.

Early response is defined as agreement between the participant and therapists about being free of PTSD symptoms, a total score below 13 (items 1–20 minus items 15, 16, 19 and 20) on the PTSD checklist for DSM-5 (PCL-5) for 2 consecutive sessions and after consultation with at least one researcher and with colleagues in weekly peer-supervision. In case a participant in the sequential condition is free of PTSD symptoms at the first follow-up measure (T1), the participant will still start with the PTSD treatment until these criteria of early response are reached.

#### Therapists and training

Therapists in this study are experienced and licensed master’s level psychologists. All therapists are trained in all three methods to avoid therapist effects. Before participation in the trial, therapists attended a two-day training in PE provided by Agnes van Minnen, a 5 day training in EMDR provided by Erik ten Broeke and a two-day training in ImRs provided by Arnoud Arntz. After finishing the trainings, all therapists had to pass a treatment integrity assessment with pilot patients by handing in a video for each type of therapy to demonstrate their competence. During the study, all therapists receive weekly one-hour peer-supervision sessions and tree yearly two-hour supervision sessions: one with each of the abovementioned trainers. There are several highly experienced therapists in each peer-supervision group. When questions arise that cannot be solved in peer-supervision, the trainers are consulted.

#### Treatment fidelity

All treatments sessions are recorded on audio. A random selection of sessions will be rated for treatment fidelity by trained junior researchers, using fidelity rating scales that were constructed in consultation with the professionals who provided the therapist training, before the start of the trial. For each treatment type, we will determine adherence to the protocol and test whether the rated sessions contained more elements of the allocated treatment type compared to elements of the other treatment types, to test treatment differentiation. This will be done with a simple t-test or non-parametric test, depending on the distribution of the data.

#### SUD treatment

Alongside PE, EMDR or ImRs, all study participants receive regular SUD treatment. Inpatient as well as outpatient participants can take part in the study. Standard care for SUD treatment includes both cognitive behavioral therapy (CBT) [46] as well as Acceptance and Commitment Therapy (ACT) [47]. Regular outpatient SUD treatment consists of 13 individual 50-min sessions (CBT or ACT) delivered by a psychologist (MSc) or cognitive behavioural therapist (BSc or MSc), supervised by a licensed healthcare psychologist. Regular inpatient SUD treatment consists of a 12-week CBT-based therapy program, of which the patient spends the first 6 weeks in the treatment facility receiving 24-h care. Patients in SUD day treatment follow a similar 12-week therapy program, for 3-days a week, 6-h a day. Timing and content of regular SUD treatment will not be modified due to or affected by participation in the study.

### Procedure

#### Recruitment

All patients attending an intake at the Jellinek Center are screened for PTSD with the J-PTSD screening questionnaire [30]. After the intake, the intramural or extramural SUD treatment is planned according to regular procedures and usually starts between 1 to 3 months after the intake. Patients with a positive PTSD screener are invited for further assessment with the Clinician Administered PTSD Scale for DSM 5 (CAPS-5) interview, also a regular procedure that is already implemented at the Jellinek Center. If the patient meets the criteria for PTSD, the psychologist informs the patient about the study and hands over the patient information letter. The psychologist asks consent of the patient for being contacted by telephone by a researcher to receive more information about the study. Potential participants receive written and oral information about the study and are invited for an informed consent procedure and screening of in- and exclusion criteria. Eligible patients are invited for the baseline (T0) assessment. Written informed consent is obtained prior to the assessment. All reasons of potential participants to decline participation in the study will be monitored.

#### Randomization and procedure

After the baseline assessment (T0), patients are randomly assigned to PE, EMDR, ImRs or SUD treatment only (each 25% chance). All patients receive SUD treatment shortly after the baseline assessment. Patients allocated to PE, EMDR or ImRs receive PTSD and SUD treatment simultaneously (75% of the sample), whereas patients in the 3-month SUD treatment only condition receive PTSD treatment after completing 3 months SUD treatment (25% of the sample). Patients in the SUD treatment only condition are randomly allocated to either PE (33% chance), EMDR (33% chance) or ImRs (33% chance) and start with the PTSD treatment after the 3 months follow-up assessment (T1).

After randomization, only disclosure of the timing will be given to the participants. Disclosure of treatment type will be done by the therapist at the start of the first session of the PTSD treatment.

Randomization is carried out by an independent researcher from Arkin who uses a computer-generated block randomization schedule. First patients are allocated to: simultaneous PE, simultaneous EMDR, simultaneous ImRs of sequential treatment with allocation probabilities per condition of 1/4. Block sizes are 4 and 8. Second, participants randomized to sequential treatment are allocated to: sequential PE, sequential EMDR or sequential ImRs, with allocation probabilities per condition of 1/3, after completion of the 3-months follow-up (T1) assessment. Block sizes are 3 and 6. Randomization is stratified by type of care (inpatient, daytreatment or outpatient) and location (Amsterdam or Utrecht). To prevent selection bias, all researchers are blind to order of block size and will not have access to the randomization schedule.

#### Assessments

Assessments take place at baseline (T0), after 3 months (T1), 6 months (T2) and 9 months (T3). The T0 takes place within 1–2 weeks before the start of SUD treatment. Therefore, participants randomized to simultaneous SUD/PTSD treatment will receive PTSD treatment between baseline (T0) and 3-month follow-up (T1), whereas participants randomized to sequential SUD/PTSD treatment will receive PTSD treatment between 3-month (T1) and 6-month (T2) follow up. See also the flow chart in Fig. 1. All assessments are conducted by a junior researcher (MSc in Psychology), who is blind to treatment condition. In case of unblinding during an intermittent measurement, another assessor will administer the next measurement(s). All assessments consist of a 45–60 min interview-administered instrument to measure severity of PTSD symptoms (CAPS-5) and several self-report questionnaires. In total, assessments take 90–120 min to administer. All participants receive a gift voucher of €15 for each assessments to acknowledge the time and effort they provide in participating in the research. The self-report questionnaires that are administered are described in the following section and listed in Table 1.

**Table 1:** Measurement instruments at every measurement moment

	Screening measurement	T0	T1	T2	T3
**Interview**					
CAPS-5		x	x	x	x
SCID-5-CV	x				x
**Self-report**					
BSI		x	x	x	x
AUDIT		x	x	x	x
DUDIT		x	x	x	x
MATE-Q module 1		x	x	x	x
DERS		x	x	x	x
IIP-32		x	x	x	x
TRGI		x	x	x	x
TRSI		x	x	x	x
ZECV		x	x	x	x
TiC-P			x	x	x
EQ-5D		x	x	x	x
Extension of LEC-5		x	x	x	x
ITQ (part 2)		x			
CTQ-sf		x			
DES subscale from SSQ		x			
STROOP & delay discounting task		x			x

Legend: *Abbreviations*: *CAPS-5* Clinician Administered PTSD Scale for DSM-5, *SCID-5-CV* Structured Clinical Diagnostic Interview for DSM-5 disorders – clinician version, *BSI* Brief Symptom Inventory, *AUDIT* Alcohol Use Disorder Identification Test, *DUDIT* Drug Use Disorders Identification Test, *MATE-Q module 1* Meten van Addicties voor Triage en Evaluatie, *DERS* Difficulties in Emotion Regulation Scale, *IIP-32* Inventory of Interpersonal Problems, *TRGI* Trauma-Related Guilt Inventory, *TRSI* Trauma-Related Shame Inventory, *ZECV* Zelf Expressie en Controle Vragenlijst, *TiC-P* Trimbos iMTA questionnaire for Costs associated with Psychiatric illness, *EQ-5D* EuroQol, *LEC-5* Life Events Checklist for DSM-5, *ITQ* International Trauma Questionnaire, *CTQ-sf* Childhood Trauma Questionnaire-short form, *DES* Daily Emotional Support subscale. Note T0–3 are subsequently the baseline assessment, and assessments at 3 month, 6 month and 9 month follow up

Furthermore, participants and their therapists also administer self-report questionnaires during therapy sessions. Study drop-out is minimized by having the follow-up assessments organized by the junior researcher, independently of the treatment facility, inviting participants for assessments regardless of treatment completion. Reminder calls are made before each assessment.

### Instruments

#### Eligibility screening

At the eligibility screening interview, SUDs are determined with the SUD criteria of the Dutch version of the Structured Clinical Interview for DSM-5 Disorders-Clinician version (SCID-5-CV) which is called the SCID-5-S [48]. Furthermore, in and exclusion criteria are assessed. A high suicide risk is checked with the suicide criteria of the Mini-International Neuropsychiatric Interview (MINI) [49] in combination with verifying if there was a suicide attempt in the last 3 months.

#### Primary outcome measure

The main study parameter is severity of PTSD symptoms and is measured at every assessment with the Dutch translation of the Clinician Administered PTSD Scale for DSM 5 (CAPS-5). The CAPS is the gold standard in PTSD assessment. It is a 30-item structured interview, corresponding to the DSM-5 diagnosis for PTSD, to assess PTSD symptoms over the past month. The sum of items 1 through 20 provides the total severity score [50]. The Dutch translation of the CAPS-5 has a high internal consistency for the full PTSD scale and a high interrater reliability score for the PTSD symptom severity score. Internal consistency for the symptom clusters is found to be acceptable [51]. All junior researchers were trained in the use of the CAPS-5 before conducting assessments and receive ongoing supervision throughout the study. Randomly selected CAPS-5 assessments are recorded on audio and scored by other researchers to check the interrater-reliability.

#### Secondary outcome measures

*Treatment completion:* Completion of PTSD treatment (yes/no) and completion of SUD treatment (yes/no) is administered by the involved therapists.

*Brief Symptom Inventory (BSI):* The BSI is a 53-item questionnaire that measures psychological distress and is developed from the longer Symptom Checklist (SCL-90-R) with very good internal consistency and test-retest reliability. All questions are scored on a Likert scale ranging from 0 to 4 and reflects the degree of distress in the last week. Total scores range from 0 to 212. It covers nine primary symptom dimensions. On all scales, higher scores indicate higher levels of complaints [52].

*Alcohol Use Disorder Identification Test (AUDIT):* The AUDIT is a World Health Organization (WHO) initiated screening instrument for problematic alcohol use in the past year. It consists of 10 statements about the amount of drinking and problems due to drinking. Items are scores on a Likert scale ranging from 0 to 4, therefore total scores range from 0 to 40, with higher scores indicating higher levels of excessive drinking. The self-reported AUDIT is used in this study [53].

*Drug Use Disorders Identification Test (DUDIT):* The DUDIT is a sensitive and specific instrument to screen for drug-related problems. It consists of 11 items and is developed as a parallel instrument to the AUDIT. Total scores range from 0 to 44, with higher scores indicate more drug use and problems related to drug use [54].

The AUDIT and the DUDIT are originally based on alcohol and drug use during the last year. In this study, at T1, T2 and T3 patients are asked about their alcohol or drug use in the previous 3 months instead of the previous year.

*MATE-Q-NL, module 1:* The Meten van Addicties voor Triage en Evaluatie (MATE-Q-NL, module 1), is a Dutch questionnaire that measures lifetime substance use and substance use over the past 30 days [55]. Only use over the past 30 days will be used as a secondary outcome measure in this study. When people are already abstinent at the first measurement assessment, substance use over the last month of their use will be administered, to prevent a flattering effect of the baseline measurement.

*Inventory of Interpersonal Problems (IIP-32):* The IIP-32 is a short version of the Inventory of Interpersonal Problems (IIP) and consists of two sections respectively asking participants what they find hard to do (for example ‘join in groups’) and things they do too much (for example ‘get irritated’) [56]. The IIP-32 consists of 32 items scored on eight subscales, with items scored on a Likert scale ranging from 0 to 4, with higher scores indicating higher levels of interpersonal problems. Internal consistency of the Dutch translation is acceptable [57].

*Difficulties in Emotion Regulation Scale (DERS):* The DERS measures emotion dysregulation across various dimensions on a 36-item self-report scale, with all questions scored on a Likert scale from 1 to 5. Higher scores indicate higher levels of emotion regulation problems. Internal consistency of the DERS is high and test-retest reliability is good [58].

*Trauma Related Guilt Inventory (TRGI):* The TRGI is a 32-item questionnaire with a 5-point Likert scale, measuring feelings of guilt related to trauma exposure. Higher scores indicate higher levels of guilt The TRGI has high internal consistency and adequate temporal stability [59]. In this study the same index will be used as in the study of Boterhoven [60]. Beliefs of victims of their role in trauma are part of the DSM-5 diagnosis (criterium D) and also an important focus in therapy (PE as well as EMDR and ImRs).

*Trauma Related Shame Inventory (TRSI):* The TRSI consists of 24 items, which can be scored on a 4-point Likert scale. Higher scores indicate higher levels of shame. In this study the same index will be used as in the study of Boterhoven [60]. As well as guilt, also shame is included as a symptom in the DSM-5. According the TRSI shame is described as a negative and painful self-evaluation in the traumatic context and a tendency to hide and withdraw from others. The TRSI had good generalizability and also good distinction between shame and guilt and can be used in both clinical research and practice [61].

*Self Expression and Control Scale (SECS):* The Zelf Expressie en Controle Vragenlijst (ZECV) is the Dutch translation of the Self Expression and Control Scale (SECS). This 40-item scale assesses internalised and externalised anger and anger control, scored on a Likert scale ranging from 1 to 4. Higher scores indicate higher levels of anger. Psychometric properties are good [62].

*Extension of the Life Event Checklist for DMS-5 (LEC-5):* The original LEC is a self-report questionnaire measuring exposure to possibly traumatic events. It was developed together with the CAPS-5 [63].

The LEC-5 is based on the DSM-5 and assesses exposure to 17 potentially traumatic events. According to the DSM-5, an event is considered traumatic if there is actual or threatened death, serious injury or sexual violence [64]. In the current study, several items have been added to the original LEC-5 to assess whether participants have experienced (recent) victimization and/or (recent) perpetration. Only recent victimization and recent perpetration will be used as a secondary outcome measure, the questions regarding lifetime trauma experiences will be used as a baseline assessment to identify the nature and extent of trauma experiences.

*PTSD Checklist for DSM-5 (PCL-5):* The PCL-5 is a 20-item self-report measure of PTSD symptoms according to the DSM-5 with a Likert scale ranging from 0 to 4. The total score is an index of PTSD symptom severity and has strong internal consistency and test-retest reliability [65]. This questionnaire will be assessed at the start of each therapy session to evaluate changes in PTSD symptoms during treatment.

*Session checklist:* A session checklist is used to gather retrospective information on the use of 12 categories of substances, on gambling, and the use of psychiatric medication in the days prior to the session. This information is registered at the start of each treatment session. This information will be used to keep up with modifications in substance and medication use during therapy.

#### Cost-effectiveness and cost-utility

An economic evaluation is conducted alongside the randomized trial and is performed according to the intention-to-treat principle. We will evaluate the relationship between costs and health outcomes of treatments. We will perform both a cost-effectiveness analysis with PTSD symptoms as effect measure and a cost-utility analysis using QALYs.

*EuroQol (EQ-5D-5L):* The EQ-5D-5L is a self-report instrument comprising five dimensions (mobility, self-care, daily activities, pain/discomfort, and depression/anxiety), that is administered at T0 and all follow-up measures to calculate health utilities [66]. Health utilities will be calculated from the EQ-5D-5L scores by using the Dutch tariff to obtain preference-based utilities [67].

*Trimbos/iMTA Questionnaire for costs associated with Psychiatric illness (TiC-P):* The TiC-P is used to measure healthcare uptake and productivity losses (absenteeism and presenteeism) between baseline and last follow-up measure (T3). Healthcare costs per participant will be determined by multiplying healthcare uptake by unit costs as described in the Dutch costing guideline [68].

#### Measurement - other study parameters

*International Trauma Questionnaire (ITQ, part 2):* The Internationale Trauma Vragenlijst (ITV) is the Dutch translation of the International Trauma Questionnaire (ITQ). It is a self-report questionnaire that measures PTSD and complex PTSD (CPTSD) and is based on the International Classification of Disease (ICD-11), in which (in contrary to the DSM-5) the diagnosis complex PTSD is also included. Part 1 of the questionnaire consists of 9 questions involving PTSD and part 2 consists of 9 questions regarding CPTSD, scored on a Likert scale ranging from 0 to 4 with higher scores indicating higher levels of complaints [69]. In this study, only part 2 is assessed.

*Childhood trauma Questionnaire – short form (CTQ-sf):* The CTQ-sf is a 28-item self-report questionnaire with questions on a Likert scale ranging from 1 to 5, that measures child abuse and neglect and can be used to identify individuals with a history of maltreatment. It has good validity and reliability and is the most widely used instrument to assess childhood trauma [70, 71].

*Daily Emotional Support (DES) subscale from Social Support Questionnaire of Transactions (SSQT):* The DES subscale of the SSQT consists of 5-items and is included in the current study to measure emotional support [72].

*Neurocognitive assessment:* Impulsivity and cognitive interference are measured by a computerized emotional STROOP task including substance-related, PTSD-related, and neutral words (Inquisit) and delay discounting is measured using a computerized version (MATLAB) [73]. Neurocognitive measures of impulsivity, like delay discounting, and cognitive/attentional interference (such as the STROOP task) have been found to relate to a higher chance of treatment dropout and earlier relapse, in several substance use disorders [74–76]. We therefore included a delay discounting task and an (emotional) STROOP task in this study, as in SUD/PTSD patients, these neurocognitive measures may influence treatment response and/or relapse.

*Baseline characteristics:* Demographic characteristics, treatment history, and patient preference of type and timing of PTSD treatment are administered.

*Treatment characteristics:* The number of sessions of PTSD-treatment attended and the number of sessions of SUD-treatment attended are administered.

*Therapist characteristics:* Therapist working experience and therapist preference of type and timing of PTSD treatment are administered.

### Data analyses

All analyses will be conducted on an intention-to-treat basis. To compare types and timing of PTSD treatment, (Generalized) Linear Mixed Models ((G)LMMs) will be used to model the primary and secondary outcome measures, with the underlying distribution (e.g., normal, gamma, (negative) binomial) depending on the type of outcome variable and its distribution. Next to these analyses based on classical statistics, a (hierarchical) Bayesian approach will be used to model the outcome parameters so that the relative evidence for the null-hypothesis (no differential effects) and the alternative hypothesis can be estimated. Prior distributions have been specified.

#### Primary outcome measure

The primary outcome measure will be the continuous variable PTSD symptom severity, as measured with the CAPS-5 at all follow-up assessments. Additionally, based on the same CAPS-5 assessments we will evaluate the dichotomous variables loss of PTSD diagnosis (no longer meeting criteria for PTSD) and full remission of PTSD (CAPS-5 score < 20), as these dichotomous variables are easy to interpret and are needed to calculate the number needed to treat. Most secondary outcome measures are continuous variables, with the exception of treatment completion of PTSD treatment and completion of SUD treatment, which are dichotomous variables.

##### Objective 1: comparison separate PTSD treatments with SUD treatment only condition at 3-month follow-up

The 3 treatment conditions will be compared with SUD treatment only condition (PE vs SUD; EMDR vs SUD; ImRs vs SUD) at 3-month follow-up (T1 measure) with a linear regression model. Baseline CAPS-5 score will be included as covariate.

##### Objective 2: comparison timing of treatment, regardless of treatment type

The effect of timing of PTSD treatment on the primary outcome will be evaluated (simultaneous vs sequential) with (G)LMMs. Baseline CAPS-5 score will be included as covariate and timing of treatment as fixed effect. Overall effects will be evaluated, as well as between-group differences at separate follow-up time points (6-months and 9-months after baseline), by adding time and an interaction between group and time to the model. The intercept will be treated as a random effect.

##### Objective 3: head-to-head comparison PTSD treatments, regardless of timing of treatment

The 3 treatment conditions will be compared head-to-head (PE vs EMDR; PE vs ImRs; EMDR vs ImRs) with (G)LMMs. Baseline CAPS-5 score will be included as covariate and treatment type as a fixed effect. Overall effects will be evaluated, as well as between-group differences at separate follow-up time points, by adding time and an interaction between group and time to the model. The intercept will be treated as a random effect.

#### Secondary study parameters

Secondary outcome parameters will be analysed with (G)LMMs, with the underlying distribution depending on the type of outcome variable and its distribution, in accordance with the above described analyses.

##### Exploration of interaction effects between timing and type of PTSD-treatment

We expect to have insufficient statistical power to detect statistically significant interaction effects between timing and type of PTSD-treatment. However, descriptive statistics of PTSD symptom severity (CAPS-5 scores) and treatment completion rates of all randomized groups will be presented (PE-simultaneous; EMDR-simultaneous; ImRs-simultaneous; PE-sequential; EMDR-sequential; ImRs-sequential), to explore whether there is anecdotal evidence for the existence such interaction-effects, which would be relevant for future research.

##### Cost-effectiveness

An economic evaluation will be conducted alongside the randomized trial and will be performed according to the intention-to-treat principle. Using a societal perspective, we will evaluate the relationship between costs, as measured with the TiC-P – and health outcomes of treatments at 9 months follow-up. A (secondary) health care perspective analysis will also be done. We will perform both a cost-effectiveness analysis with PTSD symptoms as effect measure and a cost-utility analysis using QALYs, based on the EQ-5D-5L.

### COVID-19 adjustments

Due to the COVID-19 outbreak in March 2020, the Jellinek treatment centers were entirely closed for new treatments for 6 weeks. During this time, inclusion of new participants was temporarily halted. The informed consent procedure and eligibility interview was conducted through video calling, however the baseline assessment was temporarily halted for participants who met the inclusion criteria. All assessments for patients that were already included were conducted by phone in line with lockdown restrictions. All included participants were informed about the changes in the procedure by a participant information letter about the modifications of the procedure. During these assessments an extra questionnaire on COVID-19 effects was temporarily included. This questionnaire consisted of 20 questions involving the effects of the Covid-19 outbreak on personal daily life and (mental) health. This questionnaire was designed by The Netherlands Study of Depression and Anxiety (NESDA) cohort study.

Patients in all treatment arms who had already started with treatment received the remainder of both their SUD and PTSD treatment through video calling during lockdown. No adjustments were made to the treatment protocols. For patients whose SUD treatment was postponed because of the COVID-19 measures, the PTSD treatment was also postponed until the COVID-19 measures were lifted and the SUD treatment as usual started.

Six weeks after the start of the first Covid lockdown new patients were included again, however they received their treatment as well as the assessments through video calling from the start. Baseline assessments also restarted, although measurements were done either by video calling or face-to-face.

When a range of lockdown measures lifted over the summer in 2020 and face-to-face sessions were possible again at the treatment center, all PTSD treatments were continued with face-to-face sessions. Since then, new participants all receive their entire treatment face-to-face at the location. However, when a therapist or patient is not allowed to come to the treatment facility center due to COVID-related symptoms, the session is delivered through video calling. For all sessions and assessments it is registered whether the session or assessment has been done by video calling or through a face-to-face session.

## Discussion

Completion of this RCT will provide more knowledge about the relative effectiveness of three treatment strategies for PTSD and their optimal timing in a population of patients with co-occurring SUD and PTSD. We will directly compare the effects of a PTSD treatment that is well-established for SUD nonetheless leads to high drop-out percentages (PE) and one treatment which is well-established for PTSD without SUD co-morbidity (EMDR) and one promising treatment for PTSD (ImRs). Furthermore, alcohol and drug use, treatment completion, psychological complaints, interpersonal problems, emotion regulation, trauma related emotions and cost-effectiveness of the three interventions will be examined. In addition, we will examine whether simultaneous or sequential treatment of SUD and PTSD is most effective.

### Methodological considerations

Strengths of this study are that we expect to include a cultural and socioeconomic diverse sample, since the participating centers are located in large cities and both intramural and extramural patients are included. A representative sample will be acquired by applying a minimum of exclusion criteria. The relatively long follow-up measurements of 9 months will provide insights in the long-term effects of the therapies. All therapists will be trained in all methods to prevent bias.

An important limitation of this study is the risks of high dropout rates at follow up measurements. Previous studies in this patient group have reported high assessment dropout rates [11]. We expect that patients might decide to drop out of the study once they hear they are allocated to a timing or type of treatment that differs from their personal preference. To limit this possibility, only disclosure of the timing will be given after randomization and disclosure of the treatment type will be done at the start of their first session of PTSD treatment, so that the therapist can motivate the patient for this kind of treatment. In addition, reminder calls are made before each assessment. Another limitation is that the power might be too low to detect small differences in effectiveness between types of PTSD treatment. Finally, due to the COVID-19 outbreak, temporarily adjustments in both treatment as well as measurements had to be made. Besides effects of these changes on measurements, there is possibly also a general effect of COVID-19 and the lockdown measures on the well-being of the participants. Therefore, at measurements during this period, it may be difficult to distinguish effects of treatment changes from general effects of this period.

### Conclusion

Patients with both PTSD and SUD have a high burden of disease. Currently, there is evidence that a simultaneous PE treatment is effective to treat PTSD in patients with co-occurring SUD, although effects are small, treatment dropout rates are high, and there is reluctance among therapists to offer PTSD and SUD treatment at the same time. Treatment guidelines for co-occurring PTSD and SUD can be improved based on the findings of this study, which may improve treatment outcomes of patients with this common comorbidity.

## Data Availability

The datasets and materials used or analyzed during this study will be available at the completion of this study from the corresponding author on reasonable request.

## Abbreviations

ACT: Acceptance and Commitment Therapy
AMC: Amsterdam academic Medical Center
AUDIT: Alcohol Use Disorder Identification Test
BSI: Brief Symptom Inventory
CAPS-5: Clinician Administered PTSD Scale for DSM-5
CBT: Cognitive Behavioral Therapy
CPTSD: Complex PTSD
CTQ-sf: Childhood Trauma Questionnaire-short form
DERS: Difficulties in Emotion Regulation Scale
DES: Daily Emotional Support subscale of the SSQT
DSM-5: Diagnostic and Statistical Manual of Mental Disorders
DUDIT: Drug Use Disorders Identification Test
EQ: EuroQol
EMDR: Eye Movement Desensitization and Reprocessing
(G)LMMs: (Generalized) Linear Mixed Models
IIP: Inventory of Interpersonal Problems
ImRs: Imagery Rescripting
ITQ: International Trauma Questionnaire
LEC-5: Life Events Checklist for DSM-5
MATE-Q: Meten van Addicties voor Triage en Evaluatie
MINI: Mini-International Neuropsychiatric Interview
NESDA: Netherlands Study of Depression and Anxiety
NTR: Nederlands Trial Register
PCL-5: PTSD Checklist for DSM-5
PE: Prolonged Exposure
PTSD: Post-Traumatic Stress Disorder
RCT: Randomized Controlled Trial
SCID-5-CV: Structured Clinical Diagnostic Interview for DSM-5 disorders – clinician version
SCL-90: Symptom Check List revised
SECS: Self Expression and Control Scale
SSQT: Social Support Questionnaire of Transactions
SSRI: Selective Serontonin Reuptake Inhibitor
SUD: Substance Use Disorder
TiC-P: Trimbos iMTA questionnaire for Costs associated with Psychiatric illness
TRGI: Trauma-Related Guilt Inventory
TOPA: Treatment Of PTSD and Addiction
TRSI: Trauma-Related Shame Inventory
WHO: World Health Organization
ZECV: Zelf Expressie en Controle Vragenlijst
