# Correction: Treating posttraumatic stress disorder in substance use disorder patients with co-occurring posttraumatic stress disorder: study protocol for a randomized controlled trial to compare the effectiveness of different types and timings of treatment

**DOI:** 10.1186/s12888-022-04092-x

**Published:** 2022-07-19

**Authors:** Sera A. Lortye, Joanne P. Will, Loes A. Marquenie, Anna E. Goudriaan, Arnoud Arntz, Marleen M. de Waal

**Affiliations:** 1 Arkin Mental Health Care, Jellinek, Amsterdam Institute for Addiction Research, Amsterdam, The Netherlands; 2 Amsterdam UMC, Department of Psychiatry, University of Amsterdam, Amsterdam, The Netherlands; 3 Amsterdam Public Health Research Institute, Amsterdam, The Netherlands; 4 Department of Clinical Psychology, University of Amsterdam, Amsterdam, The Netherlands

**Correction: BMC Psychiatry 21, 442 (2021)**

**https://doi.org/10.1186/s12888-021-03366-0**

Following publication of the original article [1], the authors would like to correct the text under the heading **Sample size**. The updated text is given below.

**Sample size**

First, the required sample size was calculated to explore differential effectiveness between active PTSD treatments in reducing PTSD symptoms, regardless of timing of PTSD treatment. Three pairwise comparisons will be conducted between the active treatment arms (ImRs vs. PE, ImRs vs. EMDR; PE vs. EMDR). Due to the exploratory nature of this objective, an alpha of 0.05 and power of 0.80 are acceptable. Patients in the SUD treatment only condition will be randomly allocated to PE, EMDR or ImRs after SUD treatment and therefore are included in comparisons of the active treatment arms. To detect a medium effect size (d=0.50) in a pairwise comparison between two active treatment arms, with alpha=0.05, power=0.80 and within-person correlation coefficient=0.60, 52 participants are needed per arm. To make three pairwise comparisons 3 * 52 = 156 participants are needed for this comparison.

Second, the required sample size was calculated to compare the timings of PTSD-treatment (simultaneous vs. sequential), regardless of type of PTSD-treatment. The allocation ratio between sequential and simultaneous PTSD treatment is 1:3. To be able to detect a medium effect size (d=0.45) in a comparison between two arms, with an alpha=0.05, power=0.80 and within-person correlation coefficient=0.60, 41 participants are needed in the sequential arm and 123 participants are needed in the simultaneous arm. In total, 41 + 123 = 164 participants are needed for this comparison. Finally, we expect 20% of the participants to drop out of the study, therefore in total 205 participants will be included in the study.

The original article [1] has been corrected.
